# The changing landscape of infective endocarditis in the Netherlands

**DOI:** 10.1007/s12471-015-0752-z

**Published:** 2015-09-22

**Authors:** B.L.J.H. Kietselaer

**Affiliations:** Departments of Cardiology and Radiology, Maastricht University Medical Center, PO Box 5800, 6202 AZ Maastricht, The Netherlands

Despite advances in the diagnosis and treatment of infective endocarditis (IE), the in-hospital mortality of IE has remained unchanged at about 20 %. Subgroups of patients such as those with prosthetic valve IE, IE associated with congenital heart disease or caused by certain microorganisms such as *Staphylococcus aureus* are at a much higher risk for complications, and may have one-year mortality rates of up to 60 % [1]. These high mortality rates, in combination with the advent of antibiotics, have led to the recommendation of antibiotic prophylaxis in guidelines since the early 1950s. Lately, increasing debate concerning microbacterial resistance due to possible overuse of antibiotics and lack of evidence supporting the use of antibiotic prophylaxis, has led to a change in policy in favour of restriction of antibiotic prophylaxis. The 2008 National Institute of Health and Clinical Excellence (NICE) guideline for IE has been cause for debate, advocating complete cessation of the use of antibiotic prophylaxis altogether. However, up to 20 % of UK physicians admit to prescribing antibiotic prophylaxis in very high-risk groups [2]. The situation in the UK may therefore be rather similar to the Netherlands, where 2008 European Society of Cardiology (ESC) and national guidelines recommend antibiotic prophylaxis use in high-risk patients, during high-risk procedures.

In the current issue of the Journal, Krul et al. [3] have shown a gradual increase in the incidence of IE in a large non-academic hospital in the Netherlands. They have started the registry of cases in parallel with the introduction of the new guidelines for the prevention of endocarditis in the Netherlands. The design of the study may have caused under-registration of the occurrence of IE, as only the diagnosis treatment code (DBC) was screened for IE. Patients suffering from multiple disorders in intensive care environments may thus have been missed. Interestingly, the authors show that the increase of IE is mainly due to increase in prosthetic valve endocarditis, thus a population that received antibiotic prophylaxis to prevent IE according to ESC and Dutch guidelines. One could conclude from these figures that the increase in IE incidence is due to a higher prevalence of predisposing factors for IE rather than more restrictive prescription of antibiotic prophylaxis. This is in line with several recent studies. Finally, improving on larger studies with a similar design [4], the authors were able to retrieve the causal microorganism in all IE cases. They show that microorganisms typically found in mucosal infections caused the majority of IE cases.

The notion that IE may be caused by bacteria from the mouth was suggested over 100 years ago [5]. Dental procedures have been pointed out as a source for bacteraemia causing IE. However, several large studies compared dental extraction with daily activities such as tooth brushing, and found that brushing teeth can cause bacteraemia in up to 32 % of cases [6]. Their data suggest that bacteraemia due to simple daily activities (including chewing food) may occur hundreds of times more often than bacteraemia due to dental procedures, or other medical procedures. This again calls the use of antibiotic prophylaxis for IE prevention into question. Rather, prevention of longstanding low-grade infection seems prudent.

Krul et al. [3] suggest a large-scale registry to further elucidate mechanisms underlying IE increase in the Netherlands. Such efforts have been undertaken in international consortia such as the International Collaboration for Endocarditis. Other successful approaches have employed population-wide entry of all medical records in research databases such as the Rochester Epidemiology Project in Olmsted County, USA. With the recent introduction of mandatory International Classification of Disease (ICD-10) registration of all patients within the Netherlands, a Dutch national registry is possible. While registry of disease will improve disease understanding, prevention of IE could be most effective by education of our patients at risk for IE. Dental hygiene and prevention of caries and parodontitis are an integral part of the Dutch guidelines, and should be discussed with any patients at risk. In addition, patients suffering from alarm symptoms such as ongoing fever despite antibiotic treatment, or symptoms indicative of heart failure or thrombotic events, should seek medical attention. Patient education, and thereby patient empowerment, could be one of the most important tools to improve patient survival in IE.

## Funding

None applicable to this subject.
